# Alcohol audio-visual content in formula 1 television broadcasting

**DOI:** 10.1186/s12889-018-6068-3

**Published:** 2018-10-03

**Authors:** Alexander B Barker, John Britton, Bruce Grant-Braham, Rachael L Murray

**Affiliations:** 1 UK Centre for Tobacco and Alcohol Studies, Division of Epidemiology and Public Health, University of Nottingham, Clinical Sciences Building, City Hospital, Nottingham, NG5 1PB UK; 2 University of Bournemouth, Dorset House, Talbot Campus, Fern Barrow, Poole, BH12 5BB UK

## Abstract

**Background:**

Exposure to audio-visual alcohol content in media is associated with subsequent alcohol use among young people. In 2016 Heineken launched its global Formula One (F1) partnership and had a significant brand presence at a number of 2017 F1 race events. We have measured the extent to which Heineken and other alcohol content appears in a sample of the first 6 races broadcast in the UK during the 2017 F1 Championship.

**Methods:**

We used 1-min interval coding to quantify alcohol content in all broadcast footage, including advertisement breaks.

**Results:**

Alcohol content occurred in all of the races shown and in 41% of all advertisement breaks in the programming. The most prominent content was alcohol branding, occurring in 39% of race footage intervals. Alcohol branding consisted mostly of billboard advertisements or branding on the side of cars or racing suits with *Heineken* and *Johnnie Walker* being most prominent. Alcohol branding was shown in race footage from countries where alcohol promotion is prohibited. All of the race footage was broadcast on Channel 4 on a Sunday, with start times ranging from 12:35 to 18:45.

**Conclusion:**

Audio-visual alcohol content, including branding, was highly prevalent footage of 2017 F1 races broadcast during peak viewing times in the UK. This content is likely to be a significant driver of alcohol consumption among children and adolescents.

## Background

Alcohol consumption in the UK is the 8th highest in Europe [1], was responsible for at least 6813 deaths in the UK in 2015 [2], and cost the NHS £3.5 billion in 2013–2014 [3]. Preventing alcohol morbidity and mortality is therefore a clear public health priority.

There is strong evidence that exposure to advertising or other alcohol audio visual content (AVC) in the media increases subsequent use in adolescents [4–7]. While alcohol content on TV is regulated by the Office of Communications (OfCom) code ( [8], Rule 1.10) and must not be condoned encouraged or glamourized in programmes likely to be viewed by children, pitch-side or other advertisements at the venue of televised sporting events are not regulated or controlled due to the Advertising Standard’s Agency’s definition of advertising [9].

The 2016 Formula One (F1) Championship was viewed by a global audience of 390 million people, including 21.8 million in the UK [10]. In June 2016, Heineken announced a global partnership with F1, allowing the brand to receive a *‘significant presence’* at F1 race events [11] and thereby potentially generate significant alcohol AVC. Furthermore, several races in the F1 calendar take place in countries where alcohol advertising is prohibited, and it is unclear whether alcohol advertising appears during coverage of these races in the UK.

The aim of this study was to explore the amount and type of alcohol AVC in broadcast footage of the F1 Championship. We have therefore quantified alcohol AVC in a sub-set of races from the 2017 F1 Championship.

## Methods

The 2017 Formula 1 Championship took place between 26th March 2017 and 26th November 2017 and featured races in 20 countries. Due to the time required to code a single race, we have sampled the first six races from the 2017 F1 Championship. These races were chosen to be representative of the Championship as they were diverse in terms of; their location, occurring in countries across 3 continents (Europe, Asia, and Australia); their alcohol restrictions, with two events in countries where alcohol advertising is prohibited (Bahrain and Russia); the anticipated level of alcohol advertising, with one race (China) where Heineken was the ‘Title Partner’ (that is, the name Heineken appeared in the title of the event as: ‘Formula 1 2017 Heineken Chinese Grand Prix’); and their broadcast type, three of the races (Bahrain, Russia and Monaco) were broadcast live, and three as highlights, a delayed telecast in which the broadcaster, Channel 4, broadcasts selected footage from the race rather than the race in its entirety, this included both pre and post-race footage as well as footage from the race. This is representative of the Championship as a whole as 50% of races shown in the 2017 championship were broadcast live.

The race footage, including advert breaks, was recorded, viewed and coded using the 1-min interval period method as previously described [12–15]. The method includes recording the presence of audio-visual alcohol content every 1-min in following categories:

- *Actual Use*: Use of alcohol onscreen by any character (for example, seeing a person actually consume alcohol on screen
- *Implied Use*: Any implied alcohol use without any actual use on screen (for example, seeing a person holding a drink/bottle of alcohol, but not actually consuming alcohol).
- *Other Alcohol Reference*: The presence onscreen of alcohol or other related materials (for example, bottles or beer pumps not currently in use or advertising materials)
- *Brand Appearance*: The presence of clear and unambiguous branding (for example, when a brand is identified on screen). *Alcoholic and 0% or low alcoholic products were treated separately.*
- *Any Alcohol Content:* Any of the above.

Multiple instances of the same category in the same 1-min period were considered a single event; however, if two instances of different categories occurred, these were recorded as two different events. Appearances which ran into consecutive 1-min periods were coded as separate events.

## Results

In total, 970 1-min coding intervals were viewed and coded from the first six races of the championship, 833 from race footage and 137 from advertisement breaks. The six races took place in Australia, China, Bahrain, Russia, Spain and Monaco. Three of the races (Bahrain, Russia and Monaco) were broadcast live, and three as highlights. In the UK there are no differences in regulation between alcohol advertisements in live broadcasts and highlight broadcasts [8]. All of the race footage was broadcast on Channel 4 on a Sunday, with start times ranging from 12:35 to 18:45. On average each race, including advert breaks, lasted 2 h 41 min (Range = 2 h 21 min – 2 h 50 min).

### Any alcohol content

A total of 382 (46%) 1-min intervals of racing, and 18 (13%) 1-min advertisement intervals contained any alcohol content, with occurrences in all six races and 41% of advertisement breaks (Table 1).

**Table 1:** Alcohol AVC content by type and location

Type of content	During races N (%)	During advertisements N (%)
Any Alcohol Content	382 (46)	18 (13)
Actual Use	12 (1)	2 (< 1)
Implied use	39 (5)	14 (10)
Other Alcohol References	366 (44)	16 (12)
Brand Appearance^a^	325 (39)	13 (8)
Alcohol branding	294 (30)	13 (8)
Heineken 0.0 branding	153 (16)	0 (0)

^a^N may not add up to the total due to 1-min intervals containing both alcohol and Heineken 0.0 branding

### Actual use

Alcohol consumption was limited to consumption of a sparkling drink which may be presumed to be alcohol on the winner’s podium and occurred at the end of all six races in a total of 12 (1%) 1-min intervals. There were only 2 1-min intervals (< 1%) from advertisement breaks depicting alcohol consumption (involving Thatcher’s cider and Coors Light beer) (Table 1).

### Implied use

Implied alcohol use was observed in 39 (5%) race intervals, predominantly featuring a sparkling drink (presumed to be champagne) being sprayed from the winner’s podium.

### Other alcohol references

Alcohol references, predominantly involving billboard and car advertising, occurred in 366 race intervals (38% of the total intervals from races). ‘When you drink, never drive’ billboards appeared in 71 (10%) of race intervals.

Alcohol references also occurred in 16 (12%) 1-min intervals from advertisement breaks, and predominantly comprised implied use during advertisements for alcohol brands (Table 1).

### Brand appearances

**Fig. 1:**
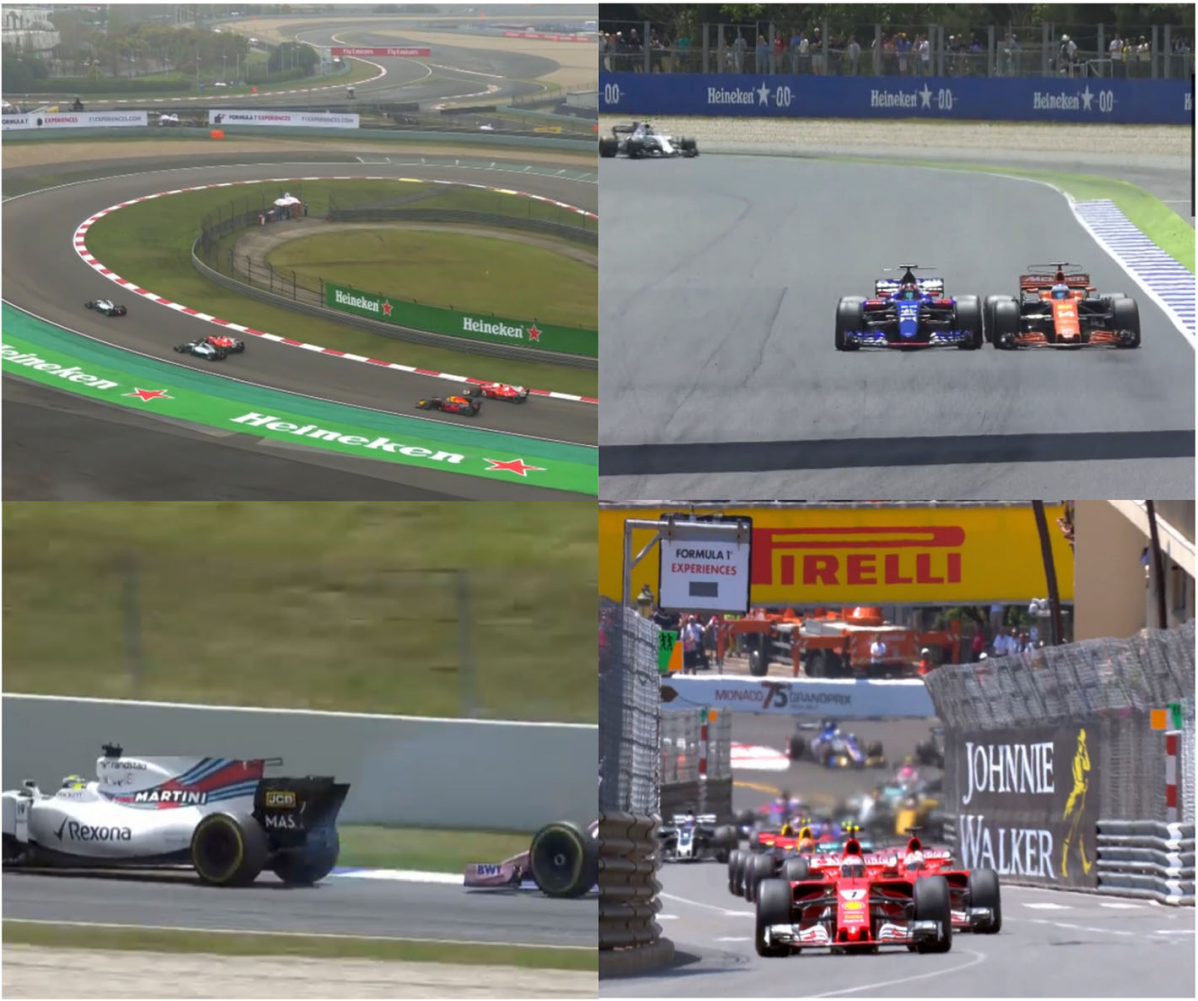
Examples of alcohol branding in F1 broadcasts (Images used with permission from Formula 1)

**Fig. 2:**
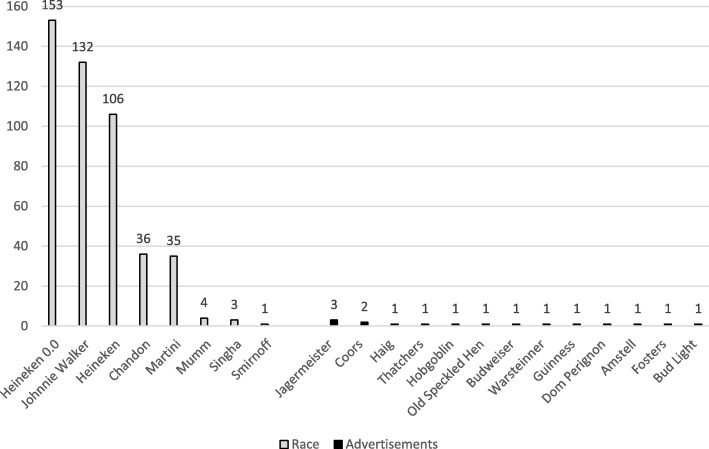
Brands identified in race footage and advertisements

Alcohol product branding occurred in 325 (39%) race intervals and consisted mostly of billboard advertisements or branding on the side of cars or racing suits. The most common brands observed overall were *Johnnie Walker,* which occurred in 132 intervals and appeared 874 times and *Heineken,* which appeared in 106 1-min intervals and appeared on screen 800 times. *Heineken 0.0,* a non-alcoholic lager which shared the *Heineken* brand name and imagery, occurred in 153 1-min intervals and appeared 972 times (Fig. 1). Alcohol branding occurred in 13 (8%) advertisement break intervals, with the most common brand being *Jägermeister* (Table 1). A list of all the brands observed is presented in Fig. 2.

The prevalence of brands shown during races differed based on the race venue. Johnnie Walker appeared in only 21 intervals before the Monaco grand prix, the final event we coded; during the Monaco grand prix Johnnie Walker was seen a total of 845 times across 111 1-min intervals. The non-alcoholic brand, *Heineken 0.0* was seen only during the Spanish Grand Prix and the Monaco Grand Prix. Both Russia and Bahrain prohibit the promotion of alcohol, however, *Martini* branding was observed during the Bahrain race on *Williams* cars, and *Heineken* had a large billboard advertising presence at the Russian race.

#### Differences between race venues

The amount of alcohol content and alcohol branding was highest in Monaco GP and lowest in the Bahrain GP (Table 2).

**Table 2:** Comparison of alcohol content between race venues

	Number of 1-min intervals (% proportion of race intervals)					
	Australia	China	Bahrain	Russia	Spain	Monaco
Any Alcohol Content	110 (44%)	73 (63%)	20 (13%)	57 (38%)	69 (59%)	126 (81%)
Alcohol Use	4 (2%)	1 (1%)	1 (1%)	3 (2%)	1 (1%)	3 (2%)
Implied Use	15 (6%)	10 (9%)	4 (3%)	7 (5%)	10 (9%)	3 (2%)
Other Alcohol References	104 (42%)	72 (63%)	17 (11%)	55 (36%)	63 (54%)	126 (81%)
Brand Appearance	87 (35%)	72 (63%)	8 (5%)	50 (33%)	31 (26%)	122 (78%)

## Discussion

This study demonstrates that audio-visual alcohol content, including branding, was present in 100% of races and 41% of advert breaks, and occurred on average once every 3 min in this sample of 2017 F1 championship race broadcasts. The majority of this content featured *Heineken* and *Johnnie Walker* branding.

Some alcohol producers associate their advertising with responsible drinking alibi messages, messages about responsible drinking while still promoting the alcohol brand by containing alcohol product trademarks. Alcohol industry messages about responsible drinking have been shown to be ambiguous and can serve a subtle public relations function, engendering goodwill with potential consumers of the brand while promoting brand preference and product consumption [16, 17]. Heineken’s 5-year F1 sponsorship has been heavily criticised for*, “*linking a popular motor sport to a significant cause of avoidable physical, mental and social harm and more specifically one of the major killers on our roads, drink driving” [18]. This global campaign incorporates Heineken’s characteristic red star and green branding with a prominent *“When You Drive, Never Drink”* message [19]. Indeed Heineken has said that it will use F1 to promote this campaign, supported by ambassador Sir Jackie Stewart [20]. Heineken has also said that it will use the sponsorship as an opportunity to promote their zero-alcohol *Heineken 0.0* brand, and indeed this was the most commonly advertised product in this study, despite only appearing in 2 of the 6 events (Spain and Monaco)*.* This argument, however, distracts from the more important point that these broadcasts deliver extremely high levels of alcohol brand advertising, or brand association through *Heineken 0.0*, in weekend television that is likely to be seen by a substantial audience of children. Potential consumers, and particularly young people, may well not distinguish advertising for alcoholic and non-alcoholic *Heineken* products due to the distinctive Heineken logo and star used on advertising billboards for both brands (see Fig. 1). The F1 Championship featured branding from a number of competing alcohol companies, it is likely that these alibi messages are part of a larger integrated marketing campaign including constant brand images, TV ads, sponsorships, and images [21, 22].

Channel 4 state that they ‘*abide*’ by the Office of Communications (Ofcom) Broadcasting Code [8], which considers factors that determine whether a programme should be shown including *‘the likely number and age range of children watching, taking into account school time, weekends and holidays’* [23]. The F1 championship races were broadcast on a Sunday afternoon, a time when children are likely to be watching what their parents are watching [24]. The Ofcom code also states that *‘Before the watershed, …the misuse of alcohol must never be condoned, encouraged or glamorised and scenes showing such material should generally be avoided unless there is editorial justification’* [8]. All of the F1 races featured scenes of drivers celebrating their win by drinking and spraying champagne, a traditional celebration in F1 [25], the consumption and use of alcohol in celebration could be glamourising alcohol use. However, we do note that in Islamic countries this may not be the case in deference to religious prohibitions as a sparkling non-alcoholic drink is used instead of champagne.

Alcohol content and brand prevalence differed between event venues, with the Monaco event containing the most alcohol content and the Bahrain event containing the least. Different countries have different restrictions on alcohol advertising [26] which may explain the differences we found, however, alcohol advertising did occur in events in countries where alcohol advertising is prohibited, raising concerns that alcohol brands are bypassing country specific regulations in the F1 races. We only examined footage broadcast in the UK and it is possible that alcohol branding was removed from country specific broadcasts, in future this should be explored. Similarly, only 6 of the 20 races broadcast were coded due to the time required to code each race. While these races were diverse in both their location and their alcohol restrictions, future studies should explore content in the entire F1 Championship to account for variations between races. Sales data for prevalent brands could also be explored to identify if the presence of branding in such events led to an increase in sales. Channel 4 also broadcast qualifying race and pre and post-event programmes, in this content analysis we focused on the broadcast containing the actual race as specified by Channel 4, because of this it is likely that this content analysis underestimates the amount of alcohol AVC shown in the F1 Championship.

## Conclusion

Alcohol content shown on TV, including alcohol advertisements and branding, is known to have an effect on the uptake of alcohol use in young people [27]. The large amount of alcohol content broadcast during the F1 races are likely to be seen by children due to the time of the broadcasts, and this exposure could lead to subsequent alcohol use. Future lawmakers also need to be aware of the arguments being used when alcohol producers are promoting their low or alcohol-free products. These often share the same branding as the producer’s full alcohol product and therefore the non-alcoholic products are providing an alibi. Restrictions on alcohol AVC during sporting events are needed to protect children and adolescents from this avenue of alcohol advertising.

## Abbreviations

F1: Formula one
OfCom: Office of communications
